# Nanomaterial-based encapsulation of biochemicals for targeted sepsis therapy

**DOI:** 10.1016/j.mtbio.2025.102054

**Published:** 2025-07-04

**Authors:** Zhiwei Li, Bin Luo, Yisheng Chen, Lingling Wang, Yezi Liu, Jintong Jia, Mengsi Chen, Shuting Yang, Haojun Shi, Lihua Dai, Lei Huang, Changmin Wang, Jia Liu

**Affiliations:** a Clinical Laboratory Center, People's Hospital of Xinjiang Uygur Autonomous Region, No. 91 Tianchi Road, Urumqi, Xinjiang, 830001, China; b Fujian Key Laboratory of Toxicant and Drug Toxicology, Medical College, Ningde Municipal Hospital, Ningde Normal University, Ningde, China; c Ningde Clinical Medical College, Fujian Medical University, Ningde, Fujian Province, China; d Department of Vascular Surgery, Ningde Municipal Hospital, Ningde Normal University, Ningde, Fujian Province, China; e Department of Neurosurgery, Department of Physiology and Pharmacology, Department of Neurosurgery and Anesthesiology, School of Medicine, Loma Linda University, Risley Hall, Room 219, 11041 Campus Street, Loma Linda, CA, 92354, USA; f Shihezi University School of Medicine, North 2nd Road, Shihezi, Xinjiang, 832000, China; g Faculty of Chinese Medicine and State Key Laboratory of Quality Research in Chinese Medicines, Macau University of Science and Technology, Macao Special Administrative Region of China; h Emergency and Intensive Care Unit, Shidong Hospital affiliated to University of Shanghai for Science and Technology, Shanghai, China; i Department of Molecular Cell and Cancer Biology, University of Massachusetts Medical School, 55 Lake Avenue North, Worcester, MA, 01605, USA; j Precision Medicine Center, People's Hospital of Xinjiang Uygur Autonomous Region, No. 91 Tianchi Road, Urumqi, Xinjiang, 830001, China

**Keywords:** Nanomaterials, Sepsis, Targeted drug delivery, Nanocarriers, Precision medicine

## Abstract

Sepsis is a life-threatening condition characterized by complex pathophysiology, including dysregulated immune responses, excessive inflammation, and oxidative stress, often leading to multi-organ failure and high mortality rates. Traditional therapies, such as antibiotics and anti-inflammatory agents, face significant limitations due to issues like drug resistance and inadequate immune modulation. Recent advancements in nanotechnology have introduced innovative solutions by leveraging nanomaterial-based encapsulation systems for bioactive substances, including antioxidants, anti-inflammatory agents, and immunomodulators. These systems enhance the stability, bioavailability, and targeted delivery of therapeutic agents, addressing critical challenges in sepsis management. Key nanocarriers, such as lipid nanoparticles, polymer-based systems, and plant-derived exosomes, offer controlled release mechanisms and precise targeting capabilities, enabling superior therapeutic outcomes. Moreover, nanomaterials' multifunctional properties allow for simultaneous modulation of oxidative stress, inflammation, and immune responses, presenting a synergistic approach to sepsis therapy. However, challenges persist, including the potential toxicity of nanomaterials, scalability of production, and hurdles in clinical translation. This review comprehensively explores the transformative potential of nanotechnology in reshaping sepsis treatment strategies, highlighting its role in advancing precision medicine and its prospects for overcoming current therapeutic barriers.

## Graphical abstract

This study systematically illustrates the mechanisms by which nanotechnology is applied in the delivery of bioactive substances, inflammation regulation, immune modulation, and disease treatment, particularly in sepsis. The image covers the extraction of natural bioactive compounds, followed by the enhancement of their stability, targeting efficiency, and bioavailability through nanomaterials. It also highlights the role of nanocarriers in gene editing, drug delivery, and immune modulation. Additionally, the image addresses the challenges and future directions of nanotechnology, such as clinical translation, toxicity control, and large-scale production. Overall, the figure emphasizes the vast potential of nanotechnology in modern medicine and personalized therapies.

**Figure:**
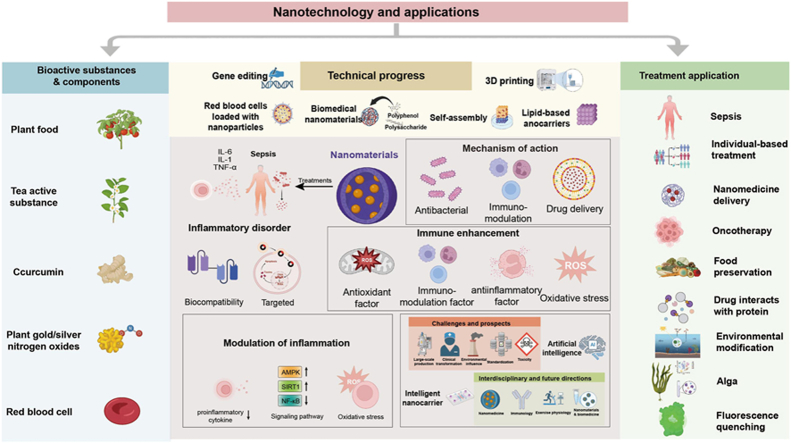

## 1 Introduction

Sepsis is a systemic inflammatory response syndrome caused by infection, which, in severe cases, can lead to organ failure and death [1]. It has become one of the leading causes of death worldwide [2]. It is characterized by a complex interplay of pathophysiological processes, including excessive inflammation, immune dysregulation, and oxidative stress, which often lead to multi-organ failure and high mortality rates. The pathogenesis of sepsis involves the initial excessive activation of the immune system, resulting in the release of pro-inflammatory cytokines such as Interleukin-1 beta (IL-1β), Interleukin-6 (IL-6) and Tumor Necrosis Factor-alpha (TNF-α). This hyperinflammatory phase can damage healthy tissues and organs [3]. Despite significant progress in early diagnosis and treatment strategies in recent years, clinical management of sepsis still faces numerous major challenges [4]. Traditional treatments primarily rely on antibiotics, but the growing problem of antibiotic resistance severely limits their effectiveness. For instance, the growing problem of antibiotic resistance has severely compromised the efficacy of antibiotics, as evidenced by studies showing that infections caused by multidrug-resistant pathogens such as methicillin-resistant *Staphylococcus aureus* (MRSA) and extended-spectrum beta-lactamase (ESBL)-producing gram-negative bacteria often lead to poor patient outcomes despite high-dose antibiotic therapy [4]. Additionally, conventional anti-inflammatory treatments lack targeted delivery capabilities, potentially exacerbating immunosuppression and increasing the risk of secondary infections. Furthermore, immune dysregulation accompanying sepsis, particularly excessive inflammatory responses, often leads to organ damage and multi-organ failure [5]. Recent clinical studies have shown that therapies targeting cytokines, such as anti-inflammatory cytokines and checkpoint inhibitors, have achieved certain therapeutic outcomes. However, these therapies face challenges such as limited efficacy and side effects [6,7]. Therefore, developing new treatment strategies, especially those that can precisely modulate inflammation and immune function, has become an urgent task [8].

**Fig. 1:**
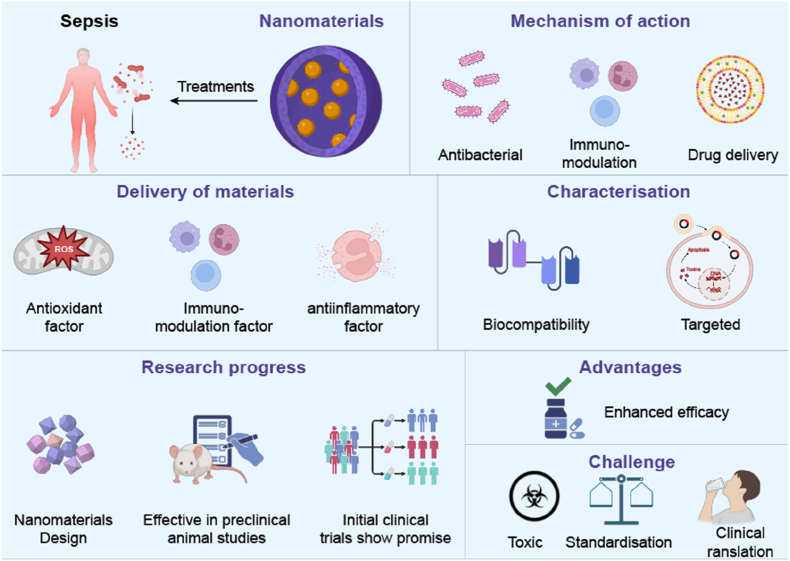
Overview of nanomaterial-based strategies for the treatment of sepsis. This figure summarizes the therapeutic potential of nanomaterials in treating sepsis. They offer antibacterial, immunomodulatory, and drug delivery benefits, targeting oxidative stress, immune dysregulation, and inflammation. Their biocompatibility and targeting ability support safe use. Research shows promising preclinical and early clinical outcomes. Despite their advantages, challenges like toxicity, standardization, and clinical translation remain.

The rapid development of nanotechnology has brought new opportunities to solve the above-mentioned problems [9,10]. Nanomaterials (with sizes ranging from 1 to 100 nm) have shown great potential in the biomedical field due to their unique physical and chemical properties, such as high specific surface area, adjustable size/morphology, and easy surface functionalization, especially in drug delivery, immune engineering, and anti-inflammatory therapy [11,12]. Chemically modifying nanomaterial surfaces (e.g., with specific ligands) enables active targeting of specific cell types or diseased tissues. This significantly improves drug targeting precision, reduces off-target effects, enhances stability and bioavailability, ultimately boosting efficacy and lowering toxicity to overcome limitations of traditional therapies [[13], [14], [15]]. As shown in Fig. 1, nanocarriers can accurately guide drugs or bioactive substances to target sites through surface engineering design, and optimize their effects through controllable release mechanisms [16]. In diseases involving immune disorders such as sepsis, nanomaterials not only serve as efficient delivery tools, but also often have the ability to regulate immune cell functions (such as macrophage polarization and T cell activation), providing possibilities for comprehensive treatment [17]. Despite its broad prospects, the clinical translation of nanomaterials still faces many challenges, mainly including in vivo biocompatibility, long-term toxicity evaluation, large-scale production, and regulatory approval barriers [18,19]. Researchers are developing engineered nanocarrier systems that can precisely deliver drugs, reduce off-target effects, and minimize unnecessary toxicity, thereby optimizing therapeutic effects [20,21].

Nanotechnology has demonstrated significant potential in revolutionizing the treatment of sepsis through enhanced drug delivery, immune modulation, and anti-inflammatory effects (Fig. 1). Nanocarriers, including solid lipid nanoparticles, nanoliposomes, and polymer-based nanoparticles, have been extensively utilized in antimicrobial therapy, drug delivery, and wound healing applications [22,23]. These systems improve the encapsulation, stability, and bioavailability of therapeutic agents while enabling precise targeting, thereby enhancing drug efficacy and minimizing systemic side effects [24]. For instance, red blood cell-derived carriers can encapsulate nanomaterials and bioactive compounds to achieve site-specific drug delivery [25], increasing drug accumulation at infected or inflamed sites and maximizing therapeutic outcomes. Additionally, nanocarriers have been shown to improve drug permeability, prolong half-life, and support sustained release, especially in chronic wound healing scenarios [26].

Plant-derived bioactive compounds, such as polyphenols, flavonoids, and terpenoids, are known for their pharmacological activities but often suffer from poor bioavailability and rapid degradation in vivo, limiting their clinical applications [27]. Nanotechnology offers a promising strategy to encapsulate these phytochemicals, thereby enhancing their stability, absorption, and therapeutic duration [28,29]. For example, the therapeutic efficacy of Morinda officinalis extract—recognized for its antioxidant, antidiabetic, and antispasmodic properties—can be significantly improved through nanoformulation. Recent advances have enabled the integration of various plant antioxidants into nanocarriers for applications in disease prevention, functional foods, and anti-aging interventions [[30], [31], [32], [33]].

Beyond pharmaceutical applications, nanotechnology also offers substantial benefits in related fields such as food packaging and antibacterial surface engineering, where it contributes to improved safety and preservation [[34], [35], [36]]. As shown in Fig. 1, the incorporation of nanomaterials into food packaging materials highlights the interdisciplinary potential of nanotechnology.

Overall, the integration of nanotechnology in sepsis treatment not only enhances drug delivery and bioavailability but also allows precise regulation of immune and inflammatory responses. Future advancements, particularly those combining nanomaterials with bioactive compounds and guided by bioinformatics and experimental research, are expected to bring transformative breakthroughs in sepsis therapy and beyond [37,38].

## 2 Overview of nanomaterials in sepsis treatment

### 2.1 Multifunctional potential of nanomaterials in sepsis therapy

**Fig. 2:**
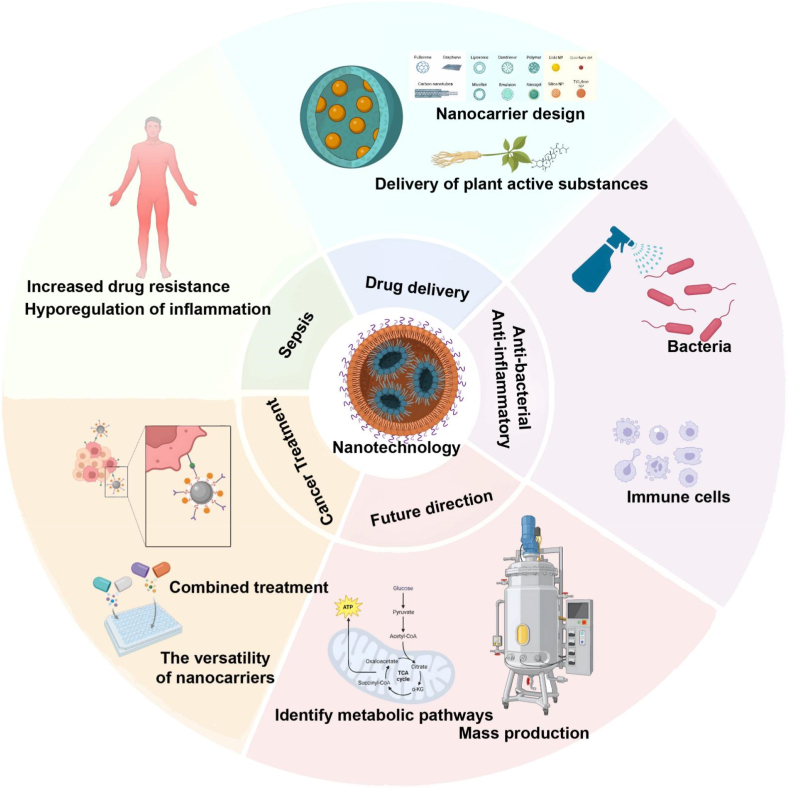
Applications and Future Directions of Nanotechnology in Therapeutics. This figure presents nanotechnology's multifaceted roles in drug delivery, antibacterial and anti-inflammatory therapy, and future developments. Nanocarriers enable targeted delivery of bioactive agents, including plant-derived compounds, enhancing efficacy and reducing systemic toxicity. Their immunomodulatory and antimicrobial functions address challenges such as drug resistance and inflammation. In oncology, nanocarriers support combination therapies with improved targeting. Future focuses include elucidating metabolic pathways and scaling production to meet clinical needs, underscoring nanotechnology's broad therapeutic potential and translational challenges.

Nanotechnology has shown great promise in revolutionizing the treatment of sepsis due to the unique physical and chemical properties of nanomaterials [39]. As depicted in Fig. 2, nanomaterials have versatile applications in drug delivery, antibacterial, and anti-inflammatory treatments [40]. Nanocarrier systems, such as those incorporating plant-derived active compounds, enhance therapeutic efficacy by precisely delivering drugs, improving drug stability, and minimizing systemic side effects [41,42]. Additionally, nanomaterials address key challenges in sepsis treatment, including drug resistance and inflammatory dysregulation, by interacting with bacterial cells and modulating immune cell functions [43]. For example, nanocarriers often utilize non-toxic, cost-effective natural polymers that combine hydrophilic and lipophilic substances with antimicrobial and antioxidant properties, optimizing drug performance while improving biocompatibility and stability [44]. This design provides a safer and more effective treatment option for sepsis patients [45]. Furthermore, nanotechnology's applications extend beyond medicine into fields like food packaging, where nanomaterials enhance antimicrobial capabilities and prolong shelf life [46]. This cross-sector application underscores the potential of nanotechnology in both infection control and sepsis treatment.

Silver nanoparticles (AgNPs) are particularly noteworthy in sepsis therapy due to their broad-spectrum antimicrobial activity and stability [47]. Research has demonstrated that the combination of AgNPs and kanamycin can inhibit pathogenic bacterial growth, with a minimum inhibitory concentration of 10–12 μg/mL, showing promise in sepsis treatment [48]. Moreover, these nanofibers can be loaded with both antimicrobial and antioxidant agents, further enhancing their therapeutic effects [49,50]. Such fibers can also be developed into sustainable packaging materials, demonstrating the interdisciplinary potential of nanotechnology. Fig. 2 highlights future research directions, including improving treatment specificity and scaling production to meet clinical demands, offering further opportunities for nanotechnology in sepsis therapy.

Nanomaterials also offer immunomodulatory benefits. For instance, lipid nanoparticles loaded with Interleukin-10 (IL-10) have been shown to reduce excessive inflammation in septic mice, improving survival rates. Nanomaterials can also promote the polarization of M2 anti-inflammatory macrophages while inhibiting M1 pro-inflammatory macrophages, thereby reducing the secretion of pro-inflammatory cytokines [51]. With their strong antibacterial activity, enhanced drug stability, and immune modulation capabilities, nanomaterials represent a promising approach for the treatment of sepsis and other infectious diseases.

### 2.2 The core mechanism of nanomaterials in sepsis therapy

(1) Antibacterial Effects

**Fig. 3:**
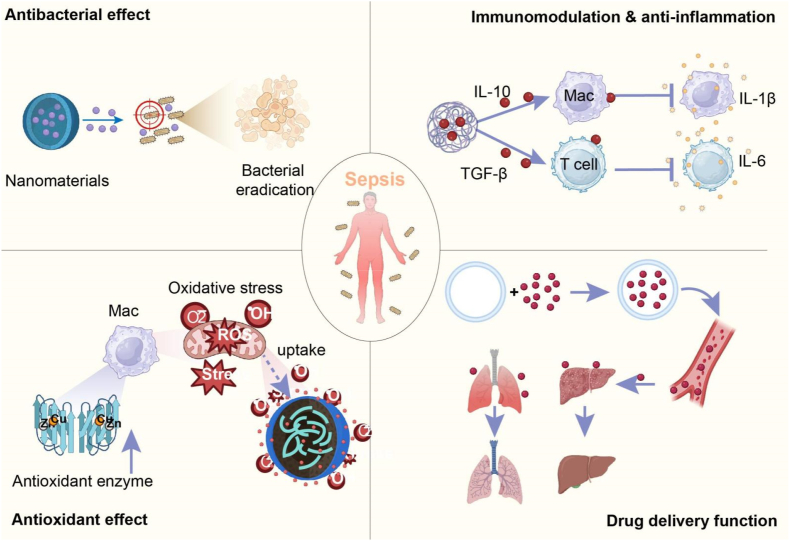
Mechanisms of Nanomaterials in Sepsis Treatment. This figure outlines the multifunctional mechanisms of nanomaterials in sepsis therapy, including antibacterial action, immunomodulation, antioxidation, and drug delivery. Nanomaterials eliminate pathogens, modulate cytokine production to restore immune balance, and reduce oxidative stress via antioxidant delivery or macrophage activation. As drug carriers, they ensure targeted delivery to vital organs, improving precision and minimizing toxicity. These mechanisms highlight the potential of nanomaterials for innovative sepsis treatment.

Nanomaterials, with their unique physicochemical properties, have demonstrated broad-spectrum and highly effective antibacterial properties, effectively inhibiting pathogen growth by destroying cell membranes, enhancing oxidative stress, and interfering with metabolic processes [52](Table 1). Surface functionalization technology further enhances the affinity and specificity of nanoparticles, thereby improving antibacterial effects [53], especially when dealing with multidrug-resistant strains [54]. Nanomaterials can exert antibacterial effects by targeting pathogens, providing a new therapeutic method for controlling sepsis. The core value of nanotechnology in antimicrobial therapy is reflected in its versatility (Fig. 2, Fig. 3). Nanomaterials provide innovative solutions for complex infectious diseases such as sepsis by interacting with pathogens and regulating immune responses [55]. Silver nanoparticles (AgNPs) are the most representative nanoantibacterial agents [56]. It mainly interacts with bacterial cell membranes, disrupting their integrity and causing content leakage. Secondly, it induces intracellular ROS accumulation, causing oxidative damage, and interferes with bacterial protein synthesis and metabolic pathways, inhibiting their growth and reproduction [57](Table 1). AgNPs exhibit broad-spectrum activity against common sepsis pathogens such as *Staphylococcus aureus* and *Escherichia coli*(*E. coli*) [[58], [59], [60], [61], [62], [63]].

**Table 1:** Core functions and mechanisms of nanomaterials.

Function	Core Mechanism	Materials/Mechanisms	Applications & Value	Reference
Antibacterial Action	Nanomaterials kill bacteria by disrupting cell membranes, enhancing oxidative stress, and interfering with metabolic processes. Surface functionalization enhances affinity and specificity.	Silver nanoparticles (AgNPs) induce ROS to damage bacterial membranes, exhibiting broad-spectrum antibacterial activity.	1 AgNPs effectively target *Staphylococcus aureus*, *Escherichia coli*, etc. 2 Green synthesis improves biocompatibility 3 Nanocomposites extend drug efficacy and reduce resistance.	Harun-Ur-Rashid, M et al. [405]
		Gold nanoparticles (AuNPs) exhibit both antibacterial and antifungal effects.		Rojas-Cessa, M. A [406].
		Nanocomposites enhance antibacterial properties.		Shehata, M. M [407].
Immunoregulation & Anti-inflammatory Action	Nanomaterials deliver anti-inflammatory factors (e.g., IL-10, TGF-β) to suppress pro-inflammatory cytokines (e.g., TNF-α, IL-1β), reducing systemic inflammation and regulating immune response.	Extracellular vesicles (EVs) deliver anti-inflammatory factors to inflammation sites.	1 EVs and MSC-Exos mitigate organ damage in sepsis. 2 Targeted therapy optimizes immune regulation.	Li, S. et al. [83]
		MSC-derived Exosomes (MSC-Exos) modulate immune responses.		Wei, P. et al. [408]
Antioxidant Action	Nanomaterials alleviate oxidative stress by scavenging reactive oxygen species (ROS), protecting cells and tissues.	AgNPs show significant free radical scavenging ability.	1 Enhanced antioxidant stability for use in pharmaceuticals and functional foods. 2 Reduces oxidative stress, protecting host tissues.	Harun-Ur-Rashid, M. et al. [405]
		Nanotechnology enhances the stability and bioavailability of natural antioxidants.		Ibrahim, S. S. S [409].

Gold nanoparticles (AuNPs) also have important antibacterial value, and AuNPs synthesized from plant extracts (such as evening primrose) are effective against both bacteria and fungi [64]. These studies have shown that AuNPs can be used for broad-spectrum antibacterial and targeted pathogen treatment [65]. By combining natural materials such as chitosan and cellulose with metal nanoparticles (Ag, Au), nanocomposites with excellent antibacterial/antioxidant properties and good biocompatibility can be prepared, providing a promising solution for sepsis treatment [[66], [67], [68], [69]]. The combination of plant-derived chemicals and nanotechnology has provided new possibilities for antimicrobial research [70]. For example, natural antimicrobial ingredients such as carvacrol and phenylpropanoids have improved stability and bioavailability after being combined with nanotechnology [71,72]. The versatility of nanotechnology and its potential for large-scale production and clinical application provide promising solutions for the treatment of sepsis [73,74].

(2) Immunomodulation and Anti-Inflammatory Effects

Excessive immune responses in sepsis treatment can lead to tissue damage and organ failure, so immunomodulation is a key strategy to improve prognosis [75]. Nanomaterials have become effective tools for immunomodulation and anti-inflammatory treatment due to their biofunctionality and design flexibility, providing a new therapeutic approach for sepsis [17]. Nanomaterials exert anti-inflammatory and immunomodulatory effects through multiple mechanisms. They reduce systemic inflammation by delivering anti-inflammatory factors such as IL-10 and TGF-β, inhibiting the expression of proinflammatory cytokines such as TNF-α and IL-1β(Table 1) [76]. Their design can target inflammatory sites and minimize systemic side effects, which is crucial for sepsis [77]. In addition, nanomaterials also regulate immune responses, especially immune cell functions. For example, they promote the generation of M2 anti-inflammatory macrophages and inhibit the differentiation of M1 proinflammatory macrophages, thereby reducing the secretion of proinflammatory cytokines [78]. Similarly, nanomaterials reduce tissue damage and lower the risk of organ failure through immunomodulation [79].

Surface engineering technology enhances the immunomodulatory function of nanomaterials [80]. Extracellular vesicles (EVs) are widely used as carriers (Fig. 2). Surface modification optimizes the targeting and stability of EVs, enabling them to precisely deliver anti-inflammatory factors [81,82]. MSC-Exos (mesenchymal stem cell-derived extracellular vesicles) can significantly downregulate pro-inflammatory cytokines such as IL-1β, IL-6, and TNF-α, and upregulate anti-inflammatory cytokines such as IL-10 and TGF-β, effectively balancing systemic inflammatory responses (Fig. 3) [83,84]. EVs technology can also effectively deliver anti-inflammatory drugs (such as RNA, proteins, and small molecules) to inflammatory sites in multiple organs [85]. Combined with imaging technology, inflammation can be monitored in real time, providing support for non-invasive treatment evaluation [86]. Future research will focus on optimizing therapeutic targeting, expanding production scale, and further combining imaging technology to provide more possibilities for clinical application. The multi-target immunomodulatory ability of MSC-Exos makes it of great value for sepsis and other inflammatory diseases [87]. Therefore, exosome-based nanodelivery systems are key to treating sepsis and inflammatory diseases due to their targeted drug delivery, immunomodulatory and anti-inflammatory properties (Table 1).

(3) Antioxidant Effects

Oxidative stress plays a key role in sepsis, with excessive reactive oxygen species (ROS) damaging pathogens and host cells, leading to tissue damage and exacerbating inflammation [88]. Therefore, the development of antioxidant strategies is crucial to alleviate inflammation and protect organ function in sepsis (Fig. 3). Nanomaterials are becoming a powerful tool to address this challenge due to their excellent antioxidant properties [89]. As shown in Fig. 2, Nanomaterials can effectively bind antioxidants and synergistically act on host cells through precise delivery and free radical scavenging functions, thereby alleviating oxidative stress and inflammation [45].

Silver nanoparticles (AgNPs) have remarkable antioxidant properties [90]. Studies have shown that the DPPH free radical scavenging activity of AgNPs is 29.55 %, which is higher than the 24.28 % of the traditional antioxidant vitamin C. The antioxidant capacity of AgNPs combined with HI extract and encapsulated in silica was further enhanced to 90.55 %. This finding suggests that AgNPs combined with plant extracts can optimize their antioxidant properties and become promising candidates for new biopharmaceutical materials [91]. Another study found that drug free tea polyphenols nanoparticles (TPNs) can improve oxidative stress status and enhance organ damage protection by clearing endogenous free radical activity, demonstrating excellent therapeutic effects in sepsis mouse models [92,93]. Antioxidants can be divided into three categories: cell defense antioxidants (such as catalase, superoxide dismutase), free radical neutralizers (such as glutathione, vitamin C), and antioxidants that repair damaged molecules. (such as proteins, DNA) [94]. Nanomaterials enhance these mechanisms, providing comprehensive protection from the cellular to the molecular level.

Nanotechnology has significantly improved the stability and bioavailability of natural antioxidants [95]. For example, nanoparticle-encapsulated Echinacea extract maintained a high retention rate of antioxidant components at high temperatures and maintained stable antioxidant activity at different temperatures [96]. This technology improves the stability and efficacy of antioxidants, and has great potential for application in medicines. In addition, nanomaterials can solve the problems of low absorption rate and high metabolic degradation rate of plant extracts and improve their bioavailability [29]. The antioxidant effect of nanomaterials is closely related to the immune response. In sepsis, the phagocytic activity of neutrophils and macrophages is impaired, leading to elevated ROS levels, which in turn exacerbate persistent inflammation and oxidative stress [97], activate matrix metalloproteinases (MMPs), and cause tissue damage [98]. By scavenging excess ROS, nanomaterials help reduce oxidative stress and inhibit inflammatory signaling pathways, protecting tissues from damage [99,100]. Nanomaterials have demonstrated significant potential in alleviating oxidative stress and inflammation in sepsis due to their versatility and high efficiency(Table 1). When combined with plant extracts and natural antioxidants, nanomaterials further enhance therapeutic effects, offering a novel approach for precise sepsis treatment. Additionally, nanoencapsulation technology improves the stability and bioavailability of antioxidants, broadening their application in sepsis and other inflammation-related diseases.

### 2.3 The common nanocarriers and optimization strategies in current sepsis therapy

Nanocarrier technology has become a key driving force for revolutionizing the treatment of sepsis. Its unique nanoscale effect endows it with the ability to improve drug bioavailability, prolong in vivo circulation time, and achieve lesion specific targeted delivery [19,101]. By precisely delivering therapeutic drugs to the site of inflammation or infection, nanomaterials can not only significantly enhance therapeutic efficacy, but also effectively reduce non-specific toxicity to healthy tissues [41]. This makes nanomaterials indispensable in treating complex infectious diseases such as sepsis, driving the continuous development of novel drug delivery systems.

(1) Natural source nanocarriers: plant exosomes and extracellular vesicles

Natural nanocarriers have received widespread attention due to their excellent biocompatibility and low immunogenicity. Plant Exosome-like Nanovesicles (PENs), as an emerging class of plant derived carriers, have shown potential in sepsis drug delivery [102,103]. PENs are rich in lipids, proteins, RNA, and small bioactive molecules, which play crucial roles in intercellular communication and maintaining homeostasis [104]. Studies show that PENs can precisely deliver regulatory factors like anti-inflammatory cytokines (IL-10, TGF-β) to mitigate excessive inflammation in sepsis, while protecting tissues from immune overactivation [102]. Efficient isolation techniques for PENs (e.g., ultracentrifugation, immunoseparation, polymer precipitation) also make large-scale clinical production feasible [105]. Extracellular vesicles (EVs), especially exosomes, are another highly promising type of natural nanocarrier [106]. EVs inherently possess anti-inflammatory, antifibrotic, and anti-apoptotic properties, which can be further enhanced through surface modification techniques to improve their targeting and drug-carrying capacity [107]. For example, EVs can precisely deliver small molecules, RNA, and proteins to inflammatory sites, inhibiting pro-inflammatory cytokines (e.g., TNF-α, IL-6) and restoring immune balance [85]. Additionally, EVs' targeting capabilities significantly reduce drug toxicity to normal tissues, enhancing treatment safety [108]. EVs also offer multifunctionality beyond drug delivery. Their integration with imaging technologies facilitates "theranostic" strategies [109]. By loading fluorescent markers or imaging agents onto EVs, real-time visualization of inflammation sites is possible [110], providing a powerful tool for evaluating therapeutic efficacy and personalizing sepsis treatment.

(2) Lipid based Nanocarrier lipid nanoparticles and liposomes

Lipid nanoparticles (NPs) have unique advantages in wound healing and local inflammation treatment due to their high surface-to-volume ratio and excellent biocompatibility [67,111]. For example, lipid NPs loaded with therapeutic agents can penetrate wound beds, directly delivering drugs to target cells [112]. Surface modification technologies can further enhance targeting, allowing preferential binding to receptors associated with the lesion, thereby improving therapeutic outcomes [113]. Nanoliposomes, another efficient drug delivery carrier, also show great potential in sepsis treatment [114]. Liposomes can efficiently encapsulate hydrophobic or hydrophilic drugs (including potent anti-inflammatory and antibacterial drugs), and improve drug enrichment and bioavailability at the lesion site by prolonging systemic circulation time and enhancing osmotic retention effect (EPR) or active targeted modification [115]. Research has shown that liposomes can effectively deliver antimicrobial peptides, anti-inflammatory molecules (such as corticosteroids), immune modulators (such as cytokine inhibitors), and even nucleic acid substances for antagonizing infection sources, calming cytokine storms, and regulating systemic inflammatory responses [116].

(3) Functional engineering strategy for nanocarriers

The core advantage of nanomaterials lies in their high designability and multifunctionality [117]. By optimizing the surface modification and structural design of nanoparticles, both drug encapsulation efficiency and release characteristics can be significantly improved [118]. For example, coating nanoparticles with natural stabilizers such as polysaccharides, proteins, or biosurfactants enhances their physicochemical stability and enables controlled release and targeted delivery of drugs [119]. These stabilizers interact with the nanoparticle surface at various binding sites, improving the carrier's particle size, solubility, and biocompatibility, which enhances drug delivery for complex diseases like sepsis [120]. As shown in Fig. 2, these strategies not only enhance therapeutic efficacy but also reduce toxicity.

(4) Combination therapy strategy based on nanocarriers

The complex pathophysiological processes of sepsis (pathogen invasion, uncontrolled inflammation, immune paralysis, coagulation disorders, organ damage) often require multi-target combined intervention. Nanomaterials are an ideal platform for co delivery or sequential delivery of multiple therapeutic drugs, such as antibiotics, anti-inflammatory drugs, and immunomodulators, supporting synergistic therapeutic strategies [121]. For instance, a nano-complex carrier platform has been developed to simultaneously deliver doxorubicin and ellagic acid for targeted lung cancer treatment [122]. Similarly, loading the non-nucleoside reverse transcriptase inhibitor efavirenz (EFV) into lactoferrin nanoparticles improves drug bioavailability and reduces toxicity [123]. These technologies can be adapted for sepsis treatment by co-delivering anti-inflammatory agents, antimicrobial drugs, and immune modulators, addressing the disease's complex pathophysiology from multiple angles.

(5) New nanocarrier Platform

As nanotechnology rapidly advances, novel carrier platforms are emerging. For instance, nanoparticles based on corn and gluten proteins show great potential in sustained-release drug applications due to their efficient encapsulation and stability [124]. Additionally, nanocarrier systems using natural bioactive molecules, such as curcumin, tannic acid, and resveratrol, significantly enhance the pharmacological activity of these compounds, providing new avenues for treating sepsis and other inflammation-related diseases [44]. The future directions for nanomaterials, as outlined in Fig. 2, also emphasize the need for large-scale production, which will accelerate the clinical translation of these advanced technologies. The integration of natural exosomes, lipid nanoparticles, and multifunctional composite carriers is paving the way for personalized and precise sepsis treatments.

## 3 Plant derived bioactive substances: active components for sepsis therapy

### 3.1 Core mechanisms of action

Plant-derived bioactive substances play an important role in the treatment of sepsis through multiple mechanisms, mainly including antioxidant, immunomodulatory and anti-inflammatory effects. First, biochemicals alleviate oxidative stress by scavenging free radicals and regulating redox balance, thereby reducing cellular damage [125]. For example, antioxidants such as sulfide, vitamin C, and vitamin E not only scavenge free radicals but also protect cellular function by interacting with key molecules involved in oxidative stress [126]. Studies have shown the potential to respond to a variety of biological responses through the regulation of specific biomolecules and the application of specific compounds [127]. Furthermore, sulfated polysaccharides extracted from Turbinaria ornata (*T. ornata*) have significant antioxidant and anticoagulant properties, further highlighting the key role of biochemicals in oxidative defense [128,129].

In terms of immunomodulation, biochemicals restore immune homeostasis by regulating immune cell activity [130]. Immunomodulatory factors such as IL-10 and TGF-β can reduce the release of proinflammatory cytokines (such as TNF-α and IL-6) and inhibit excessive immune responses (Fig. 3) [131]. These factors are particularly important in the immune imbalance triggered by sepsis. Through nanotechnology delivery, the activity and stability of these factors are enhanced, thereby improving their immunomodulatory effects [80]. In addition, biochemicals exert anti-inflammatory effects by inhibiting pro-inflammatory cytokines and modulating inflammatory signaling pathways [132]. For example, anti-inflammatory factors such as NO and TNF-α protect cells from inflammatory damage by reducing the production of proinflammatory mediators and blocking inflammatory signaling pathways such as NF-κB and p38 MAPK [133]. Plant-derived metal nanoparticles (Ag/AuNPs) can enhance the effects of anti-inflammatory factors and significantly enhance the blocking effect of pro-inflammatory signals, providing new ideas for the application of biochemicals in anti-inflammatory treatment [134].

### 3.2 Types of biochemical substances

**Fig. 4:**
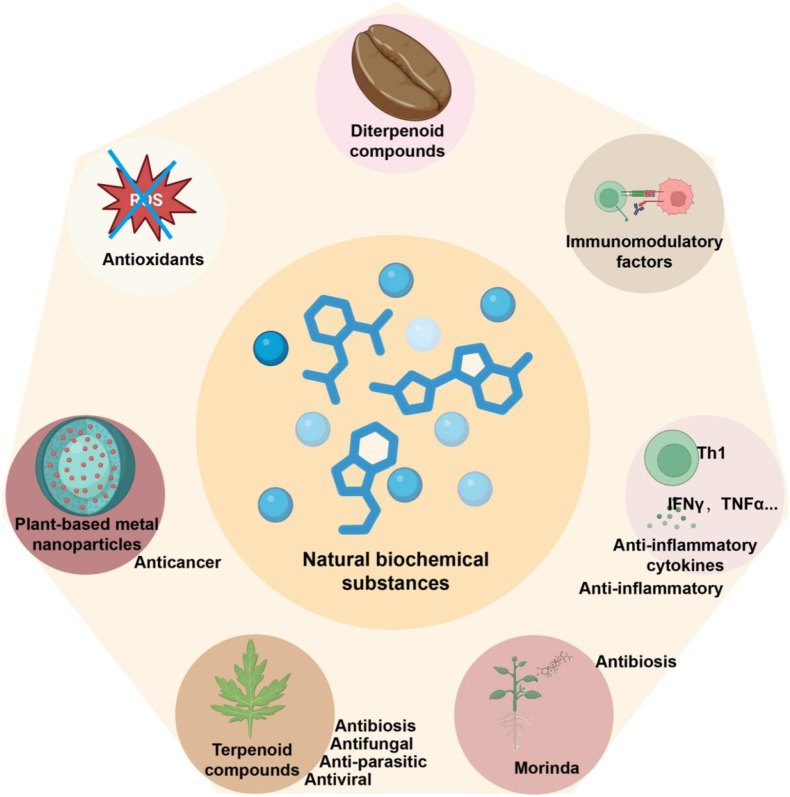
Therapeutic Roles of Natural Biochemical Substances. This figure highlights the biomedical functions of various natural compounds, including antioxidants, diterpenoids, terpenoids, immunomodulators, and Morinda-derived agents. These substances exhibit antioxidative, anti-inflammatory, antimicrobial, antiviral, antifungal, and anticancer activities. They regulate immune responses and reduce oxidative stress, offering broad therapeutic utility across multiple disease contexts.

Biochemical Substances include a variety of bioactive molecules, mainly antioxidants, immunomodulatory factors, and anti-inflammatory substances [135]. Among them, antioxidants are key components in exercise biochemical reactions, such as compounds such as sulfide, vitamin C, and vitamin E [136]. These substances protect cells by scavenging free radicals and reducing oxidative stress. In addition, diterpenoids in plants have excellent antioxidant capacity and are widely found in plants such as Arabica coffee (Fig. 4) [137]. These compounds further enhance the antioxidant effect by binding to molecules involved in oxidative stress.

Compounds such as sulfated polysaccharides extracted from algae not only have antioxidant activity but also significantly improve the performance of nanoparticles, opening up the possibility of new applications of sports biochemicals [138]. Immunomodulatory factors such as IL-10 and TGF-β can regulate immune cell function, reduce the release of proinflammatory cytokines, and restore immune system homeostasis [131]. After being combined with nanotechnology, the stability and targeting of these factors have been significantly enhanced, especially plant exosome nanoparticles (PELNs), whose low immunogenicity and high bioavailability makes them suitable for immune factor delivery and enhances their potential [[139], [140], [141]].

Anti-inflammatory factors such as NO and TNF-α play a crucial role in controlling inflammation by inhibiting pro-inflammatory signals and reducing the production of inflammatory mediators [142]. Plant-derived metal nanoparticles (Ag/AuNPs) have shown significant anti-inflammatory effects. For example, LC-AgNPs synthesized from wolfberry extract effectively blocked the inflammatory response by reducing the expression of NO and COX-2 [134]. In addition, plant-derived anti-inflammatory factors have low toxicity and are suitable for clinical application.

### 3.3 Synergistic effects of nanomaterials and biochemicals

Encapsulating plant derived bioactive substances in nanomaterials can produce significant synergistic effects. Nanomaterials protect bioactive substances from degradation, improve their solubility and absorption. For example, antioxidants encapsulated in nanoparticles have enhanced antioxidant properties and significantly improved intracellular free radical scavenging [143]. In particular, terpenoids can be combined with nanotechnology to improve their stability, penetration, and cellular activity [144]. Algae extracts combined with metal nanoparticles significantly enhanced their antioxidant and anti-inflammatory capabilities, thus broadening the application scope of sports biochemicals [145]. By functionalizing the surface of nanomaterials, targeted delivery can be achieved to the site of inflammation or infection, increasing local drug concentration and improving therapeutic efficacy. Plant-derived metal nanoparticles (such as AgNPs and AuNPs) have large surface areas and active surfaces, which can improve the efficiency of targeted drug delivery and enhance the biological effects of biochemicals (Fig. 4) [146]. The antibacterial, antioxidant, or immune regulatory functions of nanomaterials themselves overlap or complement the effects of encapsulated bioactive substances. LC-AgNPs and LC-AuNPs synthesized from wolfberry extract exhibited significant anti-inflammatory activity. The introduction of nanotechnology has also achieved the integration of "treatment and diagnosis". In sepsis treatment, this integration enables real-time monitoring of inflammatory responses and targeted drug delivery [[147], [148], [149]].

## 4 Strategies for encapsulating biochemical substances with nanomaterials

### 4.1 Overview of nanomaterial encapsulation techniques

**Fig. 5:**
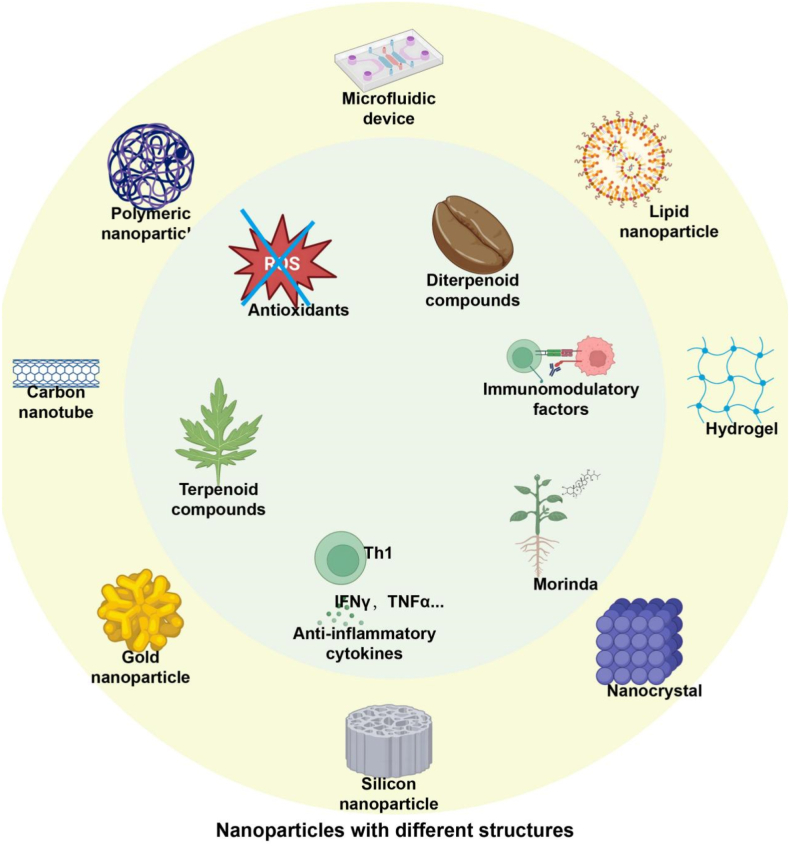
Integration of Natural Compounds with Nanoparticles. This figure illustrates the synergistic use of natural bioactive substances with diverse nanoparticle systems (e.g., polymeric nanoparticles, hydrogels, lipid nanoparticles). Such integration enhances the stability, bioavailability, and targeted delivery of therapeutic agents, enabling efficient, low-toxicity treatment. This strategy represents a promising approach for optimizing drug delivery and advancing natural compound-based therapies.

Nanomaterial encapsulation technology has become a key strategy for enhancing the stability and functionality of exercise-related biochemical substances [150,151]. By using nano-sized carriers to encapsulate active substances, this approach improves their stability, solubility, and activity in the body [29]. Techniques like solvent evaporation, co-precipitation, and gelation create efficient delivery systems for antioxidants and immune factors [152]. Nanoparticles prepared via these methods boost the targeting and biocompatibility of biochemical substances, significantly improving therapeutic outcomes for complex diseases such as sepsis. As shown in Fig. 5, various nanoparticle structures, including polymer nanoparticles, carbon nanotubes, and lipid nanoparticles, enhance the stability and targeted delivery of natural biochemical substances, supporting advanced therapeutic applications [153]. Surface functionalization optimizes interactions with immune cells and delivery pathways, improving the effectiveness of targeted therapy [154]. This is achieved through chemical modification and functional molecule design, which enhance cell affinity and nanocarrier stability.

Since its inception in the 1950s, nanomaterial encapsulation technology has evolved from basic research to widespread industrial applications [155]. Initially developed to extend the shelf life of active ingredients and control their release, it is now widely used in food science, pharmaceuticals, and biomedical fields [156]. Careful selection of nanocarrier materials, such as lipids, polymers, or metal nanoparticles, allows for the directed release of encapsulated substances while offering protection in complex biological environments [157]. For example, natural compounds like polyphenols and flavonoids, which have poor water solubility and low bioavailability, show improved stability and application potential when encapsulated in nanomaterials [158].

Despite these advances, challenges remain. Standardizing the stability and release rates of substances within nanomaterials is complex, and evaluating the bioactivity of encapsulated compounds in vivo remains a critical area of research [159]. Moreover, optimizing encapsulation techniques to accommodate diverse active substances is essential for future developments [160]. Overall, nanomaterial encapsulation technology enhances the stability and functionality of biochemical substances, offering promising solutions for treating complex diseases and laying a solid foundation for future research and applications.

### 4.2 Specific forms of nanomaterial encapsulation

Different nano encapsulation forms can be selected based on the properties and expected applications of bioactive substances. As shown in Fig. 5, various forms such as lipid nanoparticles, hydrogels, and nanocrystals significantly enhance the stability and delivery efficiency of natural biochemical substances, expanding their potential applications in drug delivery.

(1) Lipid Nanoparticles and Nanoemulsion Technology

Lipid nanoparticles (SLNs) and nanoemulsions are commonly used to encapsulate hydrophobic biochemical substances [161](Table 2). The bilayer membrane structure of liposomes enables the encapsulation of both hydrophilic and hydrophobic molecules, making them a versatile delivery system [162]. Liposomes are particularly effective in drug delivery due to their membrane-like structure, which enhances cellular uptake of drugs [163]. However, their application in the food industry is limited by low physical stability and high permeability [164]. Improving bilayer properties, such as increasing membrane fluidity and antioxidant capacity, can overcome these challenges [165]. Nanoemulsions, with droplet sizes under 100 nm, exhibit superior stability, making them ideal for encapsulating volatile or poorly soluble molecules [166](Table 2). For example, vitamin E nanoemulsions prepared with mustard oil and Tween 80 maintained stability for 15 days without coagulation [167], extending product shelf life and enhancing antioxidant properties. Compared to traditional emulsions, nanoemulsions require fewer surfactants, thus reducing production costs and increasing commercialization potential [168].

(2) Solid Lipid Nanoparticles (SLNs)

**Table 2:** Specific forms of nanocapsulation technology.

Nanocapsulation Type	Core Mechanism	Examples	Advantages	Reference
Lipid Nanoparticles & Nanoemulsions	Encapsulate hydrophobic biomolecules using lipid bilayers or emulsions.	Liposomes for drug delivery	1 Enhanced stability and bioavailability. 2 Reduced surfactant usage.	Chen, Y. et al. [286]
		Nanoemulsions for Vitamin E		Zhang, S. et al. [410]
Solid Lipid Nanoparticles (SLN)	Solid at room temperature, made from food-grade lipids, enhances solubility.	Sesame lignan encapsulation with high efficiency	1 Better physical stability. 2 Precise targeting via lipid composition.	Ma, H. et al. [255]
				Duan, W. et al. [411]
Polymeric Nanoparticles	Encapsulate hydrophobic compounds, improving bioavailability and stability.	Quercetin in chitosan/lecithin	1 Versatile preparation methods. 2 Stable under different conditions.	Zhang, S. et al. [410]
		Stable at varying pH and temperatures		Ma, H. et al. [255]
Electrospun Nanofibers	High-porosity fibers, suitable for encapsulating sensitive compounds.	Antimicrobial peptides, enzymes in fibers	1 Long shelf-life. 2 Enhanced antibacterial properties.	Hu, Y. et al. [412]
				Qu, J. et al. [413]
Nanocomposites & Smart Packaging	Nanoparticles integrated into packaging for added functionality.	Silver nanoparticles for antimicrobial packaging	1 Extended shelf life. 2 Real-time monitoring for freshness.	Harun-Ur-Rashid, M. et al. [405]
		Smart sensors for real-time monitoring		Xi, W. et al. [414]

Solid lipid nanoparticles (SLNs) are an efficient encapsulation strategy for hydrophobic substances(Table 2). Made from food-grade lipids that are solid at room temperature, SLNs offer excellent protection and significantly improve the solubility and stability of active substances [169]. For instance, SLNs encapsulating sesaminol achieved 94.3 % encapsulation efficiency and improved bioavailability [170]. SLNs are more stable than liquid lipid nanoparticles, preventing aggregation and degradation during storage [171]. They offer high structural flexibility, allowing for precise targeted delivery through modifications in lipid composition, particle size, and surface characteristics [172]. For example, incorporating phytosterols into SLNs improves solubility and antioxidant activity [173]. This technology can be applied in the food industry to extend the shelf life of functional foods and provide a reliable delivery system for hydrophobic drugs.

(3) Polymer Nanoparticles

Polymer nanoparticles, formed by polymerizing hydrophobic and hydrophilic monomers, are widely used in encapsulation for functional foods and drug delivery(Table 2). These nanoparticles not only encapsulate hydrophobic biochemical substances but also enhance biocompatibility through interactions with polysaccharides, lipids, or proteins [174]. For example, quercetin encapsulated in chitosan and lecithin shows enhanced antioxidant properties, stability, and solubility [[175], [176], [177]]. Methods such as nanoprecipitation, emulsification-diffusion, and layer-by-layer coating allow for flexible preparation, tailored to the characteristics of specific biochemical substances [178]. These nanoparticles are stable across a wide range of temperatures and pH conditions, making them suitable for both food and pharmaceutical applications [179]. Studies have demonstrated that these nanoparticles remain stable at temperatures up to 70 °C and in pH environments ranging from 3.3 to 5.0, supporting their use in diverse applications [180].

(4) Electrospun Nanofibers

Electrospun nanofibers, created using electrospinning technology, produce high-porosity structures with diameters less than 100 nm, offering an efficient method to encapsulate sensitive biochemical substances [181](Table 2). This technology is ideal for heat-sensitive and volatile compounds. Electrospun nanofiber membranes, for example, protect antimicrobial peptides and enzymes from environmental degradation, significantly extending their functional lifespan [182]. In food packaging, electrospun nanofibers provide high-performance materials with excellent mechanical properties and antibacterial activity [183]. Gelatin nanofibers containing nisin can effectively inhibit pathogenic bacteria for up to five months [184]. Crosslinking agents enhance their resistance to dissolution and physical stability, positioning electrospun nanofibers as key components in developing efficient, environmentally friendly packaging materials [185].

(5) Nanocomposite Materials and Smart Packaging

Nanocomposite materials integrate nanoparticles into conventional packaging, imparting additional functionality (Table 2) [186]. For example, incorporating antimicrobial agents, oxygen scavengers, or enzyme inhibitors into nanocomposite films can extend food shelf life and enhance safety [187,188]. The addition of metal nanoparticles, such as silver, and antimicrobial peptides, significantly boosts antimicrobial activity while improving the mechanical and barrier properties of the material [189]. Smart packaging, which integrates sensors, allows for real-time environmental monitoring [190]. Nanomaterial-based sensors can detect changes in temperature, humidity, and oxygen, alerting users to food freshness through color changes or digital signals [191]. This technology reduces waste during food transportation and storage, while improving supply chain efficiency and transparency.

### 4.3 Integration of nanomaterials with natural biochemical substances

The integration of nanomaterials with natural biochemical substances enhances the stability and functionality of active ingredients [192]. Many natural substances, such as polyphenols, flavonoids, antioxidants, terpenoids, and immune modulators, suffer from poor water solubility, instability, and low bioavailability, limiting their bioactivity [193]. As shown in Fig. 5, combining these substances with nanoparticles improves bioavailability and therapeutic effects. Nanomaterials, with their high surface area and surface modification capabilities, address these challenges by protecting the biochemical substances from degradation and optimizing their targeted delivery and release control [194]. Nanomaterials can alter the physical form of substances (such as forming amorphous forms), increase solubility and transmembrane absorption rate [195]. For example, polyphenolic compounds encapsulated in lipid nanoparticles exhibit enhanced solubility and antioxidant properties [95]. By surface functionalization (such as antibody, peptide, folate modification), nanomaterials can preferentially bind to specific cell or tissue receptors (such as receptors highly expressed at the site of inflammation), achieving active targeting [196,197].

In sepsis treatment, functionalized nanoparticles can target inflammation sites and enhance the effects of immune modulators [55]. Similarly, terpenoids encapsulated in nanoparticles show reduced volatility and improved stability, boosting their antimicrobial, anti-inflammatory, and immune-regulatory effects [198]. Nanomaterials also optimize the release patterns of natural substances. Design stimuli responsive carriers (pH sensitive, enzyme sensitive, ROS sensitive) that trigger drug release in specific microenvironments at the target site, increase local concentration, and reduce systemic exposure. Flavonoids, prone to degradation in the gastrointestinal tract, can be encapsulated to delay release, improving bioavailability [199]. Nanoemulsions and nanofibers are used for volatile essential oils, reducing volatility and enhancing solubility, thus improving their functionality in food preservation and antioxidants [200]. The use of liposomes, nanocapsules, and polymer nanoparticles not only stabilizes active substances but also expands their applications [201,202]**.** Combining bioactive substances with functional nanomaterials to generate synergistic therapeutic effects. For example, plant extracts loaded with silver or gold nanoparticles exhibit significant anti-inflammatory and anticancer effects [203]. Garlic-loaded silver nanoparticles reduce inflammatory factor expression, while goji berry-loaded silver nanoparticles show cytotoxicity against breast cancer cells [151]. This combination preserves the original functions of bioactive substances while enhancing their therapeutic effects, offering new opportunities for treating complex diseases.

In conclusion, nanomaterial integration with natural biochemical substances offers great potential for improving stability, bioavailability, and functionality. This strategy overcomes traditional limitations, with implications for sepsis and cancer treatment, as well as functional foods and health management. Advancements in nanotechnology will further expand these strategies across various fields.

### 4.4 Functionalization and improvement strategies for nanomaterials

(1) Surface Functionalization

Surface functionalization is crucial for improving nanomaterials' compatibility with biological systems and enabling targeted delivery. Nanomaterials can be surface-modified using various strategies to enhance their stability, targeting ability, and biocompatibility, Common surface modification techniques include, polymer coating, ligand conjugation, antibody conjugation, cell membrane coating, and stimuli-responsive modification [204]. Functionalization with polymers such as polyethylene glycol (PEG) can reduce nanoparticle aggregation and prolong circulation time by inhibiting nonspecific protein adsorption [205]. For example, PEGylation of gold nanoparticles (AuNPs) has been shown to enhance their stability and reduce immune recognition, thereby increasing their circulation time in the bloodstream [206]. Additionally, nanomaterials can be conjugated with targeting ligands such as antibodies, peptides, or small molecules to achieve specific targeting to sites of infection or inflammation [207]. For instance, AuNPs conjugated with anti-ICAM-1 antibodies have demonstrated selective binding to endothelial cells during inflammation, enabling targeted delivery of anti-inflammatory agents [[208], [209], [210]].

(2) Particle Size and Morphology Optimization

The size and shape of nanomaterials significantly influence their pharmacokinetics and biodistribution. By controlling these parameters, nanomaterials can be optimized for specific applications*(182*. For example, smaller nanoparticles (e.g., <100 nm) generally exhibit better tissue penetration and cellular uptake, which is beneficial for targeting deep tissues and cells [211]. Conversely, larger particles (e.g., >200 nm) may be more suitable for applications where prolonged circulation time is desired [212]. The shape of nanoparticles also affects their behavior in biological systems. Spherical nanoparticles typically exhibit enhanced stability and reduced clearance rates, while rod-shaped or discoidal nanoparticles can offer better targeting efficiency due to their anisotropic properties [213]. For example, rod-shaped gold nanoparticles have been shown to exhibit higher photothermal conversion efficiency, which can be utilized for localized hyperthermia treatments in sepsis [214].

(3) Material Composites and Multifunctional Integration

Nanomaterial composites have gained significant attention for achieving multifunctionality. By integrating different types of nanomaterials, more complex functions can be achieved [215]. For example, combining magnetic nanoparticles with optical ones enables simultaneous drug delivery and photothermal therapy [215]. Embedding antimicrobial metal nanoparticles (e.g., silver) into biopolymer matrices enhances both antimicrobial activity and mechanical durability, benefiting medical diagnostics [216]. These composites also improve nanomaterial stability and controllability [217]. For instance, combining casein with liposomes stabilizes curcumin in high-temperature or acidic environments, while silica nanoparticles enhance the photostability of vitamin E [218]. This strategy expands the range of options for drug delivery systems.

(4) Emerging Functionalization Strategies

Emerging functionalization techniques, such as dynamic and biomimetic approaches, are revolutionizing nanomaterial performance. Nanomaterials can be designed to release their therapeutic payload in response to specific stimuli, such as pH, temperature, or enzymes. This stimuli-responsive release ensures that the drug is delivered only at the site of infection or inflammation, thereby maximizing therapeutic efficacy and minimizing off-target effects [219]. For example, pH-sensitive chitosan nanoparticles selectively release drugs in the tumor microenvironment, sparing normal tissues [220]. Smart nanocomposites are also being developed for food packaging to monitor freshness by detecting temperature or humidity changes [221]. Biomimetic functionalization, which mimics natural biological functions, enhances nanomaterial biocompatibility and immune evasion capabilities [222]. For instance, coating nanoparticles with cell membranes can improve their performance in cancer immunotherapy and anti-infection treatments [223]. Additionally, biomimetic approaches can improve food packaging materials, increasing mechanical strength and antibacterial properties by simulating plant cell wall structures [224].

### 4.5 Future development directions and application prospects

The development of nanomaterial encapsulation technology for bioactive substances has transformed biomedicine and food science, yet challenges remain in its widespread application [225]. Future research will focus on optimizing functional design, improving biocompatibility and safety, assessing large-scale production feasibility, and promoting interdisciplinary integration for significant breakthroughs. Functional Design and Multifunctional Integration are key areas for future progress [226]. Artificial intelligence and machine learning can predict the relationship between nanomaterial structure and function, enabling the design of more targeted carriers [[227], [228], [229]]. For example, nanomaterials responsive to disease microenvironments (e.g., tumor acidity or elevated reactive oxygen species in inflammation) can enhance treatment targeting and efficiency [230]. Multifunctional integration—combining diagnostic and therapeutic capabilities—can advance precision medicine by enabling integrated diagnosis and treatment platforms [231,232].

Biocompatibility and Safety remain critical concerns. While nanomaterials generally exhibit low toxicity, long-term safety risks are not fully understood [233]. Systematic toxicological analyses and multi-omics approaches will be essential to investigate interactions between nanomaterials and biological systems [234]. Optimizing surface characteristics to minimize toxicity is key. Biomimetic strategies, such as coating nanomaterials with biological materials like cell membranes, can reduce immune rejection and extend circulation time, improving safety [235].

Large-Scale Production and industrial application pose major challenges. Current production methods are mostly limited to laboratories, being complex and costly, which hampers large-scale implementation. Future efforts should focus on optimizing preparation techniques, such as green chemical synthesis and efficient self-assembly, to improve economic viability and reproducibility [236]. Additionally, the stability of nanomaterials during storage and transportation needs to be addressed to maintain functionality over time.

Smart Responsive Nanomaterials offer significant potential in food, drug delivery, and sensor applications [237]. These materials can dynamically adjust their functions in response to environmental changes, such as temperature, light, or pH. For example, smart nanosenors could monitor food freshness, while responsive drug delivery systems enable precise, stimulus-controlled drug release, improving treatment outcomes and minimizing side effects [238].

The Interdisciplinary Application of nanotechnology is expanding possibilities. The integration of materials science, biology, medicine, and computational science is driving the efficient development of advanced materials [239]. For instance, combining nanotechnology with gene editing is advancing gene therapy, offering new solutions for genetic diseases [240,241]. In food science, nanomaterials enhance stability and bioavailability and can be developed as biodegradable, eco-friendly packaging materials, addressing global plastic pollution [242].

Strengthening Policy Regulations and increasing social acceptance are vital for further nanotechnology applications. As nanomaterials become more widely used, establishing international standards and regulatory frameworks is crucial. Clear guidelines, ensuring safety and compliance, along with public education, will foster trust and create a favorable environment for commercialization [243]. Bioinformatics analysis of massive data, the strategy of inducing apoptosis of immunogenic cells through key molecular pathways is constantly innovated, bringing new hope for cancer treatment [244,245]. In conclusion, nanomaterial encapsulation technology will continue to expand, with applications extending beyond biomedicine and food science into fields such as environmental protection.

## 5 Clinical research progress on nanomaterial-encapsulated bioactive substances for sepsis

### 5.1 Design and application of nanomaterials

**Fig. 6:**
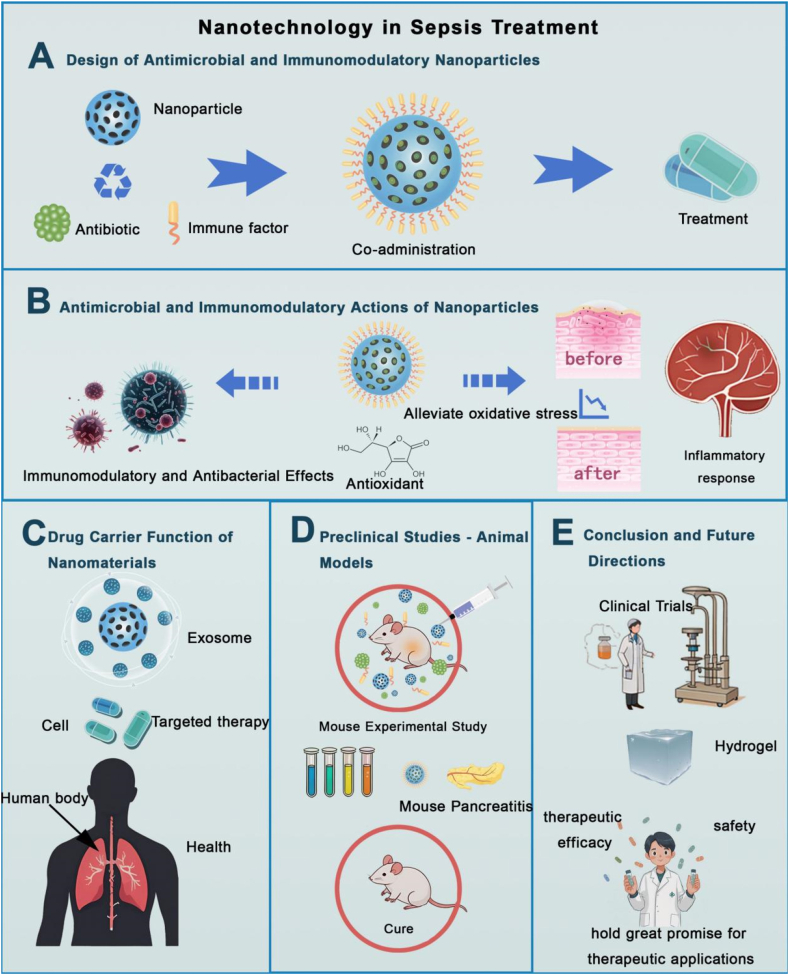
Nanotechnology-Based Approaches in Sepsis Therapy. (A) Nanoparticles are engineered with antibiotics and immune agents for enhanced effects. (B) They exhibit antimicrobial, antioxidant, and anti-inflammatory actions. (C) Nanomaterials serve as targeted drug carriers, such as exosomes for organ-specific delivery. (D) Animal studies show therapeutic potential in treating conditions like pancreatitis. (E) Future directions include clinical translation via hydrogels and other systems to improve safety and efficacy.

Nanomaterials have attracted significant attention in sepsis treatment due to their unique properties, offering innovative solutions to the disease's complex pathology [17]. By combining antimicrobial and immunomodulatory functions, nanomaterials enhance both infection control and immune regulation [80]. Nanoparticles co-engineered with antibiotics and immune factors (Fig. 6A) improve antimicrobial and immunomodulatory properties, while their ability to deliver antioxidants (Fig. 6B) helps reduce oxidative stress and inflammation.

Silver nanoparticles (AgNPs) stand out for their potent antibacterial and anti-inflammatory effects, effectively targeting both native and multidrug-resistant microorganisms while promoting wound healing [246] Innovations like near-infrared laser-activated trivalent silver nanoparticles (Tri-Ag) further boost antibacterial activity and accelerate tissue repair, offering new treatment possibilities for complex infections [247,248]. Similarly, copper nanoparticles (CuNPs) show promise in treating conditions like diabetic foot ulcers [249]. Beyond metallic nanoparticles, nanobioactive glass particles and gold nanoparticles (AuNPs) are also highly regarded for their biocompatibility and antibacterial efficiency [250]. These materials interact with cell surface proteins, such as serum albumin, and can be assessed for biocompatibility via fluorescence quenching techniques, making them valuable tools in sepsis treatment [251].

The immunomodulatory potential of nanomaterials further broadens their applications. Polyethylene glycol-amide nanoparticles (PENs) possess antibacterial and antitumor activities while modulating immune responses, such as reducing cytokines like TNF-α and IFN-γ, thereby alleviating systemic inflammation and organ damage in sepsis [252]. Natural immunomodulatory substances, such as catechins, exhibit enhanced antibacterial, anti-inflammatory, and antioxidant effects when combined with nanotechnology [253]. For instance, PEG-encapsulated EGCG nanoparticles show potent antibacterial activity and promote pathogen-induced apoptosis [254].

Hydrogels and lipid nanoparticles, known for their controlled drug release and biocompatibility, are increasingly utilized in sepsis therapies [255,256]. These materials optimize drug delivery, enhancing treatment efficiency while minimizing systemic side effects [257]. Chitosan-based materials, natural antibacterial agents, are a key focus in nanomaterials research due to their effectiveness against Gram-positive and Gram-negative bacteria, as well as their low toxicity and biodegradability [258].

Nanomaterials integrated with natural products also demonstrate considerable potential. For example, marine algae extracts combined with nanotechnology exhibit anti-inflammatory, antioxidant, and immunomodulatory effects [259]. Bee pollen-based nanomaterials possess significant antibacterial properties and promote wound healing, making them ideal for use in antibacterial dressings [260].

The design of nanomaterials is constantly evolving, surpassing single functional antibacterial activity. Multi functional nanomaterials, such as responsive polysaccharide based nanoparticles, can accurately release drugs based on changes in the pathological microenvironment. These strategies have effectively improved treatment outcomes and provided new possibilities for personalized sepsis treatment, making nanomaterials a key force in advancing research on sepsis and related diseases.

### 5.2 Applications of nanotechnology in natural biochemical substances

The nanotechnology of natural biochemical substances offers an innovative and efficient solution for the treatment of sepsis. This strategy combines the multifunctionality of natural active substances, such as antioxidants, plant extracts, and plant exosomes, with the advanced capabilities of nanotechnology, significantly enhancing their drug stability, bioavailability, and targeting efficiency. As shown in Fig. 6C, nanomaterials (such as exosomes) serve as efficient drug carriers, enabling precise delivery of natural biochemical substances to target sites, thereby improving their bioavailability and therapeutic effects.

(1) Nanotechnology of Antioxidants in Sepsis Therapy

Antioxidants are gaining attention in sepsis treatment for their ability to alleviate oxidative stress and inhibit inflammation [261]. Oxidative stress, driven by excessive reactive oxygen species (ROS), is a key pathological mechanism in sepsis, and nanotechnology further amplifies the antioxidant effects [262]. Encapsulating antioxidants in nanomaterials improves their stability and bioavailability while enhancing their ability to scavenge free radicals [263](Table 3). Composite nanofibers of α-tocopherol and β-cyclodextrin, produced via electrospinning, not only boost antioxidant activity but also enable sustained release, thereby reducing inflammation [264]. Natural antioxidants, such as carotenoids, polysaccharides, and polyphenolic compounds from microalgae, effectively scavenge ROS and protect cells from oxidative damage [265]. For example, microalgae polysaccharide-based nanoparticles demonstrate significant free radical scavenging and reduction of cell apoptosis [266]. Furthermore, co-encapsulation of astaxanthin and tocopherol in nanomaterials shows synergistic antioxidant effects, effectively reducing oxidative stress-induced apoptosis in tissues [267]. Additionally, deferoxamine (DFO) enhances wound healing by inducing HIF-1α accumulation, providing new solutions for oxidative stress-induced damage in sepsis [268]. A single atom nanoenzyme (Cu-SAzyme) with coordination unsaturated and atomically dispersed Cu-N4 sites was synthesized by simulating the electronic and structural characteristics of natural pure copper superoxide dismutase (SOD5), which can effectively treat sepsis [269].

(2) Nanotechnology of Natural Extracts in Sepsis Therapy

**Table 3:** Applications of nanotechnology in natural biochemical substances.

Application Area	Nanotechnology Functions	Key Example	Therapeutic Outcomes	Reference
Antioxidant Nanotechnology	Nanoencapsulation of antioxidants for stability and bioavailability.	α-Tocopherol and β-cyclodextrin nanofibers for sustained release and inflammation reduction.	Enhanced ROS clearance, reduced oxidative stress and apoptosis.	Feng, X. et al. [415]
Natural Extracts in Sepsis	Encapsulation of plant extracts to improve delivery and stability.	Turbinaria ornata extracts for antioxidant and anti-inflammatory effects.	Improved antioxidant, antimicrobial, and anti-inflammatory effects.	Huang, J. et al. [314]
Plant Exosomes in Sepsis	Use of plant-derived exosome-like nanoparticles for targeted delivery.	Citrus-derived exosome-like nanoparticles (ELN) for targeting and inflammation reduction.	Reduced inflammation and targeted, low-toxicity drug delivery.	Moore, A. R. et al. [103]
Future Directions	Development of multi-functional nanocarriers.	Development of polyphenol-loaded nanocarriers for combined anti-inflammatory and wound healing.	Improved stability, bioavailability, and precision in sepsis treatment.	He, Y. et al. [10]

Plant extracts have garnered attention for their broad biological activities in sepsis treatment [270]. Nanotechnology enhances the stability, pharmacodynamics, and targeting efficiency of these extracts [271]. For example, natural products from the marine algae Turbinaria ornata exhibit antioxidant, anti-inflammatory, and neuroprotective effects [272]. When nanomized, these products enhance drug stability and enable more targeted delivery for sepsis treatment [116]. Similarly, sodium alginate/polyethylene glycol (SA/PEG) scaffolds, created using 3D printing, show excellent antibacterial properties, providing new options for antimicrobial wound dressings [273]. Bee pollen, rich in polyphenols and flavonoids, demonstrates antibacterial, antioxidant, and immune-regulating properties [274]. Encapsulating bee pollen and apple cider vinegar in chitosan films enhances their antibacterial and antioxidant effects, significantly inhibiting the growth of Listeria monocytogenes, Salmonella, *E. coli*, and *Staphylococcus aureus*, while promoting tissue repair [275](Table 3). Polysaccharide-based nanoparticles, derived from sulfated polysaccharides, not only display antioxidant and anti-inflammatory properties but also promote angiogenesis and tissue repair through cytokine regulation [276].

(3) Nanotechnology of Plant Exosomes in Sepsis Therapy

Plant exosome-like nanoparticles (PELNs) are natural carriers with excellent biocompatibility and low toxicity, offering unique advantages for sepsis treatment [277](Table 3). Citrus-derived ELNs, for instance, inhibit cancer cell proliferation without affecting normal cells [278]. Dandelion-derived ELNs reduce pro-inflammatory factor production and alleviate inflammation, while those from Rehmannia glutinosa effectively suppress LPS-induced inflammation. Surface modifications enable plant exosomes to achieve targeted delivery [279]. For example, PEG-modified plant exosomes (PEG-ACNVs) extend drug blood retention time and improve delivery efficiency [280]. This green, platform-based delivery system offers a promising approach for drug delivery and gene therapy in sepsis. Studies show that adjusting the size, surface charge, and other properties of exosomes can significantly improve their accumulation at inflammation sites, enhancing therapeutic outcomes [281].

(4) Comprehensive Assessment and Future Prospects

Nanotechnology applications of natural biochemical substances have revolutionized sepsis treatment by enhancing the stability, bioavailability, and targeting efficiency of active compounds [282]. Nanotechnology-based antioxidants, such as carotenoids from microalgae, hold strong potential in alleviating oxidative stress associated with sepsis by scavenging excess ROS [283]. Combining natural extracts like bee pollen and polysaccharides with nanomaterials not only amplifies their antibacterial, anti-inflammatory, and tissue repair effects but also extends their applicability through innovative technologies [284]. Furthermore, plant exosome-like nanoparticles (PELNs) provide an efficient, low-toxicity carrier for delivering natural substances, particularly in targeted delivery and gene therapy, showing great potential for future development [102]. Despite the promising results in sepsis treatment, full clinical translation of these technologies faces challenges. Future efforts should focus on optimizing nanomaterial design and preparation processes to ensure safety and stability. Additionally, developing multifunctional nanomaterials for precise delivery of antioxidants, anti-inflammatory factors, and other natural substances tailored to specific sepsis types is essential for enhancing therapeutic efficacy.

### 5.3 Nanotechnology and drug delivery systems

Nanotechnology provides significant opportunities for the innovation of drug delivery systems, particularly for treating diseases like sepsis, which present significant treatment challenges due to their complex pathology. By harnessing the unique properties of nanomaterials, drug stability, bioavailability, and targeting at disease sites are significantly enhanced. Nanotechnology further optimizes the drug release process, reduces side effects, and opens new pathways for sepsis treatment. It plays a crucial role in precision drug delivery systems, particularly through its interaction with natural biochemical substances. Lipid nanoparticles and microfluidic devices, in particular, have significantly improved targeting capabilities, supporting precise sepsis treatment (Fig. 5).

(1) Diversified Nanomaterials and Their Advantages

Nanomaterials, including nanoliposomes, polysaccharide-based nanoparticles, nanoemulsions, and exosomes, exhibit unique characteristics that enhance drug delivery and therapeutic efficacy [285,286]. Nanoliposomes, with their phospholipid bilayer structure, can encapsulate both hydrophobic and hydrophilic drugs [287]. Liposomes loaded with antioxidants, vitamins, and antimicrobial agents significantly improve drug stability and tissue penetration [288]. For example, drugs like amphotericin B and paclitaxel, when delivered via liposomes, show higher absorption and reduced systemic toxicity [289]. Polysaccharide-based nanoparticles, particularly chitosan-based carriers, are widely used in sepsis treatment for their biocompatibility and effective drug release regulation [290]. These carriers improve drug retention time at disease sites, enhancing treatment outcomes [291,292]. Similarly, nanoparticles made from gliadin and zein proteins enhance drug encapsulation efficiency and bioavailability [293]. Nanoemulsion systems, developed for phytochemicals, improve the stability of traditionally unstable plant compounds [294]. For instance, flavonoids and resveratrol, delivered via nanoemulsions, show increased in vivo stability and resistance to digestive enzyme degradation [295]. Self-nanoemulsifying drug delivery systems (SNEDDS) optimize drug efficacy; for example, thymoquinone's relative bioavailability increases by 3.87 times in SNEDDS, enhancing its anti-inflammatory and antimicrobial effects [296].

(2) Exosomes and Plant Exosome Delivery Advantages

Exosomes, with their natural nanoparticle characteristics, have emerged as promising drug delivery tools [297]. They are biocompatible, have low immunogenicity, and can carry a wide range of bioactive substances, making them suitable for multi-target sepsis treatment strategies [298]. Milk-derived exosomes, for instance, accelerate tissue repair by modulating inflammation and promoting cell migration [299]. These exosomes reduce sepsis-related damage by decreasing pro-inflammatory factor release and ROS levels [300]. Surface modifications further enhance their drug delivery targeting capabilities [301]. Plant-derived exosome-like nanoparticles (PELNs) offer environmentally friendly and biocompatible advantages as drug carriers [302]. For example, citrus-derived exosomes, through surface modification, extend drug circulation time and improve delivery efficiency [303]. Exosomes from dandelion and Rehmannia glutinosa show exceptional anti-inflammatory effects and immune regulation, providing targeted sepsis treatment potential.

(3) Development of Targeted Delivery and Responsive Carriers

Targeted delivery is a key goal of modern nanotechnology in drug delivery. By modifying nanomaterials with specific ligands, precise targeting of cells or tissues is achieved [304]. For instance, folate-modified nanomaterials bind to folate receptors, showing excellent therapeutic effects in tumor and inflammation models [305]. In sepsis treatment, this strategy increases drug concentration at inflammation sites while minimizing systemic toxicity [114]. Responsive nanomaterials, whose drug release is controlled by microenvironmental conditions like pH, temperature, or enzyme concentration, are also promising [306]. Bacteriocins combined with nanoparticles have shown impressive results against multi-drug resistant infections [307], with responsive carriers releasing antimicrobial agents selectively at disease sites to minimize healthy tissue toxicity [308]. Multifunctional nanomaterials, integrating antibacterial, anti-inflammatory, immunomodulatory, and antioxidant effects, offer multidimensional support for treating complex pathologies [309]. Metal nanoparticles (e.g., silver and copper), when combined with bacteriocins, enhance antibacterial activity and stability, overcoming degradation by proteolytic enzymes [310].

(4) Future Development and Clinical Application Potential

The integration of nanotechnology with drug delivery systems offers immense potential for sepsis treatment, yet several challenges remain for clinical application. First, optimizing nanocarrier manufacturing processes to reduce costs and ensure scalability is essential [311]. Secondly, multi-center clinical trials combined with single-cell RNA sequencing technology are needed to verify the safety, stability and long-term efficacy of these vectors [[312], [313], [314]]. In the future, combining nanotechnology with intelligent computational methods, such as AI-driven design of responsive and multifunctional nanomaterials, will provide more precise and efficient solutions for sepsis treatment [315].

### 5.4 Progress in preclinical research

Nanotechnology has shown great promise in preclinical sepsis research, particularly in enhancing drug stability, bioavailability, and targeted delivery. In murine sepsis models, chitosan-based nanoparticles delivering antimicrobial or anti-inflammatory agents significantly reduced pro-inflammatory cytokine release and enhanced anti-inflammatory factor expression [316]. This approach boosted immune function, mitigated tissue damage, and improved survival rates (Fig. 6). Metal nanoparticles, such as silver (AgNPs) and gold (AuNPs), have also demonstrated therapeutic potential by reducing inflammation and oxidative stress [317]. Notably, AgNPs promote collagen deposition and angiogenesis in chronic wound models, particularly in diabetic conditions [318].

Exosomes, natural nanomaterials, have demonstrated unique roles in preclinical sepsis studies. Milk-derived exosomes delivering anti-inflammatory molecules alleviated systemic inflammation, improved pathology, and extended survival in animal models [319]. Similarly, exosomes from crab hemolymph exhibited promising antitumor activity, inhibiting breast cancer cell proliferation while maintaining low toxicity and good biocompatibility, opening new possibilities for large-scale exosome production and clinical translation [320].

The combination of natural biochemical substances with nanomaterials further expands the potential of nanotechnology. For example, bee pollen extract (BPE) and nanoparticles showed enhanced anticancer effects in breast and lung cancer models, reducing side effects while improving drug stability and targeting [321]. In sepsis-related immunomodulation, nanotechnology combined with natural substances improved immune function and reduced tissue damage in animal models [19].

Optimizing functionalized nanoparticles is crucial for enhancing therapeutic effects. Adjusting surface charge, solubility, and size has improved nanoparticle delivery efficiency in complex biological environments [322]. Surface-modified nanoparticles targeting antimicrobial and anti-inflammatory molecules not only reduced inflammation but also improved outcomes in sepsis models by inducing immune cell remodeling [316]. Nanocomposites combining photothermal therapy with oxygen-releasing materials significantly promoted angiogenesis and accelerated wound healing in diabetic chronic wounds, highlighting nanotechnology's role in multifunctional therapies [323].

Despite these promising results, challenges remain, including large-scale production, consistency control, toxicity assessments, and long-term biological safety validation [324]. Additionally, due to sepsis's complex pathology, the effects of nanotechnology at different disease stages require further investigation. Future research should focus on optimizing nanomaterial design, exploring their in vivo mechanisms, and investigating personalized treatment options. Overall, preclinical studies suggest nanotechnology holds significant potential in sepsis treatment, particularly in targeted delivery, enhanced bioavailability, and improved therapeutic outcomes.

### 5.5 Clinical research and Future Prospects

Nanotechnology is increasingly recognized for its potential in treating complex diseases like sepsis. Preliminary clinical trials indicate that encapsulating natural biochemical substances in nanomaterials significantly enhances the bioavailability and therapeutic efficacy of various drugs [271]. For instance, capsaicin-loaded nanoparticles (NPs) increase oral bioavailability by 3.2 times compared to free capsaicin, offering a promising solution for poorly soluble drugs [325]. Chitosan-coated gold nanoparticles (C-AuNPs) demonstrated 25 times greater oral bioavailability than PEG-coated counterparts, improving drug absorption across the digestive tract [326]. These results strongly support nanotechnology's role in chronic disease treatment.

Nanoliposomes, versatile drug carriers, have shown promise in treating skin conditions like inflammation, atopic dermatitis, and psoriasis [327]. Enriched with antioxidants, they alleviate skin irritation and enhance the bioavailability of dietary supplements, helping prevent chronic diseases [29]. The oral bioavailability of nano lotion systems, such as thymol (TQ) nano lotion, has increased 3.87 times, and has shown liver protective effects, providing a new way for disease prevention and treatment [328].

Silver nanoparticles (AgNPs) have demonstrated significant potential in wound healing due to their antimicrobial properties and ability to promote tissue repair [246]. In mouse models, AgNPs reduced wound area, promoted collagen deposition, and facilitated angiogenesis while reducing local inflammation. In diabetic chronic wounds, AgNPs accelerated healing by enhancing collagen and growth factor expression [329]. However, their local use may cause mild inflammatory reactions, necessitating careful design to ensure safety.

Despite the promising clinical trial results, challenges remain in nanomaterial application. Long-term safety evaluations, particularly concerning immune responses and potential toxicity, are essential for clinical translation [18]. Additionally, large-scale production and consistency control must be optimized to reduce costs and improve feasibility. Organoid technology provides a powerful platform for studying disease pathogenesis and drug screening by constructing disease models, combining bioinformatics analysis of model data and applying network pharmacology [[330], [331], [332], [333]]. Personalized treatment strategies, supported by randomized controlled trials and multi-center studies, are needed to further validate efficacy and safety. Future research should focus on developing precise targeted delivery systems, such as combining nanomaterials with gene-editing tools like CRISPR-Cas9 for disease intervention [334]. As shown in Fig. 6E, advancing nanotechnology in clinical trials involves enhancing efficacy and safety with hydrogels and other carriers, offering more accurate solutions for complex diseases. Furthermore, exosome-based nanomaterials and natural biochemical substances with high biocompatibility will be crucial in treating complex diseases. The integration of nanotechnology, molecular biology, and clinical medicine will continue to expand, providing more efficient and safer treatment options for conditions like sepsis.

## 6 Advantages and challenges of encapsulating biochemical substances in nanomaterials

### 6.1 Advantages of encapsulating biochemical substances in nanomaterials

(1) Enhanced Drug Delivery and Targeting Efficiency

**Fig. 7:**
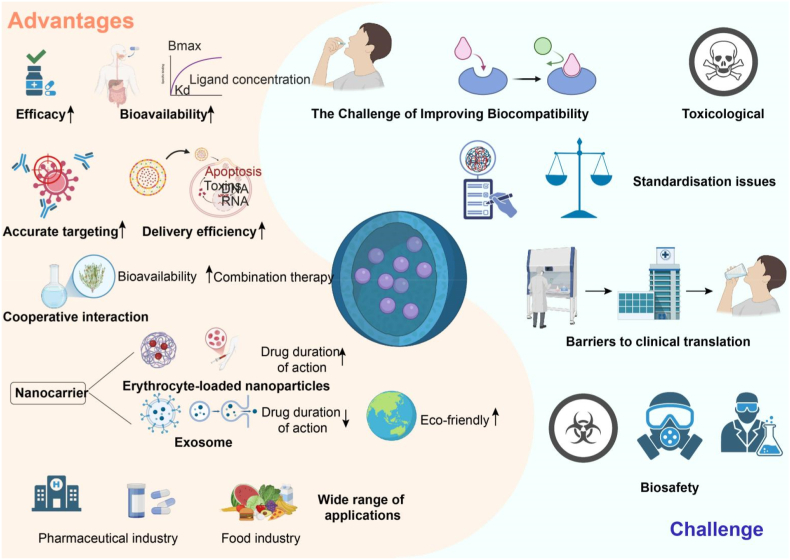
**Advantages and Challenges of Nanocarrier Drug Delivery.** This figure compares the strengths and limitations of nanocarrier systems. Benefits include improved targeting, bioavailability, and prolonged drug release, enabling combination therapies and broad applications. However, challenges such as toxicity, biocompatibility, standardization, and regulatory compliance hinder clinical translation. Addressing these issues is crucial for their broader application.

Nanomaterials greatly enhance the efficiency and targeting of drug delivery, which is crucial for treating complex diseases like sepsis. Natural biochemical substances often suffer from poor stability, solubility, and bioavailability, which nanomaterials address by acting as advanced drug carriers. For instance, red blood cell membrane-coated nanoparticles (RBC-hitchhiking) can improve drug absorption, extend drug half-life, and minimize immune clearance by targeting specific organs or tissues (Fig. 7, Table 4) [335,336].

**Table 4:** Advantages of encapsulating biochemical substances in nanomaterials for exercise.

Advantage	Functions & Example	Outcome	Reference
Enhanced Delivery Efficiency	Nanomaterials improve drug stability and targeting (e.g., RBC-hitchhiking nanoparticles).	Increased drug accumulation at target sites, extended circulation, reduced clearance.	Liu, J. et al. [23]
Improved Stability & Bioavailability	Nanoencapsulation protects bioactive compounds (e.g., catechins in nanoparticles).	Enhanced stability, bioavailability, and controlled release.	Zhang, Z. et al. [416]
Synergistic Effects	Combining nanomaterials with bioactive compounds (e.g., catechins + gold nanoparticles).	Optimized drug delivery, reduced side effects, enhanced therapeutic efficacy.	Zhang, Z. et al. [416]
Biocompatibility & Low Toxicity	Biocompatible nanocarriers for targeted delivery (e.g., exosome-based carriers).	Reduced toxicity, precise delivery, minimal off-target effects.	Li, L. et al. [417]
Eco-friendly & Sustainable	Biodegradable nanomaterials (e.g., chitosan-based carriers) for drug delivery and packaging.	Sustainable, biodegradable solutions with minimal environmental impact.	Qu, J. et al. [413]

Surface modifications, such as polymer coatings, increase targeting precision and reduce off-target effects, thus enhancing therapeutic efficacy [337]. Additionally, nanomaterials’ ability to cross biological barriers, like the blood-brain barrier, opens new avenues for treating complex diseases [338]. Stimuli-responsive materials further optimize drug release, responding to specific conditions at disease sites, which is especially beneficial in sepsis treatment [339].

(2) Improved Bioavailability and Stability

Nanomaterials significantly improve the bioavailability and stability of natural biochemical substances, which are often susceptible to degradation and poor solubility. By encapsulating bioactive molecules, nanomaterials protect them from enzymatic degradation and oxidation, thereby improving their stability and therapeutic outcomes [44]. For example, catechins encapsulated in nanoparticles maintain colloidal stability and release drugs under pathological conditions, enhancing their efficacy [340].

Nanomaterials also enhance bioavailability by modifying the physicochemical properties of bioactive molecules, facilitating their passage across biological barriers [341]. Red blood cell-loaded nanoparticles extend drug circulation time, prevent immune clearance, and improve targeting [335]. These advances allow for sustained drug release, reducing the need for frequent dosing and improving patient compliance [342,343].

(3) Synergistic Effects and Multifunctionality

Nanomaterials offer synergistic effects and multifunctionality, improving overall therapeutic efficacy. For example, combining catechins with metal nanoparticles or natural polymers like chitosan enhances bioavailability and therapeutic outcomes [344] (Table 4). Metal nanoparticles can improve antioxidant capacity, while chitosan facilitates targeted delivery, optimizing drug stability and reducing side effects [345].

Nanomaterials also enable simultaneous delivery of multiple active compounds, such as anti-inflammatory and antioxidant agents, which is particularly beneficial for treating sepsis, where immune dysregulation is a key concern [277]. Multifunctional nanomaterials, such as those responsive to acid, light, or magnetic fields, offer controlled release mechanisms for disease treatment, further expanding their applications in precision medicine [[346], [347], [348]].

(4) Biocompatibility and Low Toxicity

Biocompatibility and low toxicity are fundamental advantages of nanomaterials, making them ideal for clinical applications involving natural biochemical substances [349]. Nanomaterials, such as exosomes, exhibit low immunogenicity and high biocompatibility, delivering bioactive molecules while minimizing toxicity [350]. These materials can cross complex biological barriers, including the blood-brain barrier, while maintaining therapeutic precision [351].

Functionalized synthetic nanomaterials like chitosan also show excellent biocompatibility, enhancing drug stability and reducing long-term toxicity [352]. By targeting specific disease sites, nanomaterials can minimize non-target tissue exposure, reducing side effects and improving therapeutic outcomes [340,380]. This precision is especially important in treating sepsis, where nanomaterials help regulate immune responses and reduce systemic damage [17].

(5) Eco-Friendliness and Sustainability

Nanomaterials not only improve drug efficacy but also contribute to environmental sustainability, a key consideration in modern nanotechnology. Materials like chitosan and polylactic acid, derived from natural sources, are biodegradable and reduce environmental pollution compared to synthetic alternatives [353,354]. These materials require less energy for production and degrade quickly, aligning with green chemistry principles.

Nanomaterials also support eco-friendly applications in green pharmaceuticals, packaging, and bioremediation [44]. In drug development, nanomaterials reduce waste and enhance resource efficiency by optimizing drug release and delivery, which lowers environmental impact [355]. Additionally, nanomaterials aid in environmental remediation, helping to remove pollutants like heavy metals from water [356].

In agriculture, nanomaterials enhance biofertilizers and pesticides, promoting sustainable farming practices by reducing chemical usage and minimizing environmental contamination [357]. As nanotechnology continues to evolve, its potential for contributing to sustainability in various sectors, including renewable energy and waste recycling, will expand [358,359].

### 6.2 Challenges in nanomaterials encapsulating biochemical substances

(1) Biocompatibility, Standardization, and Quality Control

The biocompatibility of nanomaterials is crucial for their medical application, but issues such as inconsistent quality control, manufacturing variability, and the lack of standardized testing systems remain significant barriers to clinical translation [360]. Nanomaterials often struggle with poor solubility, bioavailability, and stability of encapsulated biochemical substances [361], which are essential for optimizing their therapeutic effects. While natural nanomaterials like exosomes offer high biocompatibility, large-scale production challenges and quality control issues persist [362]. Moreover, individual physiological differences complicate their interaction with biological tissues [363]. Developing standardized quality control protocols and internationally recognized testing systems is necessary to ensure the reliable application of nanomaterials across industries [364,365].

(2) Toxicity and Safety

The potential toxicity of nanomaterials is a critical concern that limits their widespread use. Some nanoparticles can accumulate in tissues, triggering inflammation or apoptosis [366,367]. For example, gold nanoparticles may negatively impact cell membranes and mitochondria, and titanium dioxide nanoparticles' toxicity is influenced by particle size and surface properties [368,369]. Strategies to mitigate these risks include developing low-toxicity biodegradable materials (e.g., plant polysaccharides and proteins) and optimizing surface modifications (e.g., PEG coatings) to minimize immune system interactions and reduce toxic effects [[370], [371], [372]].

(3) Clinical Translation and Scalability

The transition from laboratory research to clinical application faces challenges related to technical complexity and high costs. Large-scale production of nanomaterials requires high-precision, stable processes that often incur significant expenses [373]. Furthermore, more research is needed on the metabolic pathways and pharmacokinetics of nanomaterials in vivo [374]. Regulatory approvals and ethical considerations also complicate clinical translation, as nanomaterials require extensive safety testing and long-term monitoring [414]. The diverse functional properties of nanomaterials further challenge regulatory agencies in evaluating their overall safety and efficacy [375].

(4) Environmental and Biological Safety

The widespread use of nanomaterials also poses environmental risks, including potential pollution of water and soil from nanoparticles released during production and usage [376]. Certain nanoparticles can disrupt the metabolic functions of aquatic organisms or inhibit plant growth [377]. Furthermore, nanoparticles may accumulate in the food chain, posing risks to higher trophic organisms [378]. Developing biodegradable, eco-friendly nanomaterials, such as those derived from natural polymers or agricultural waste, can reduce environmental pollution [379]. Research into the migration and degradation behaviors of nanomaterials in the environment is crucial for identifying and mitigating potential ecological risks [380].

While nanomaterials encapsulating biochemical substances offer promise in sepsis treatment, their application is hindered by challenges related to biocompatibility, toxicity, standardization, scalability, and environmental impact [381]. Future research must focus on optimizing nanomaterial design and production processes, establishing comprehensive safety and quality control systems, and developing sustainable, eco-friendly alternatives.

## 7 Future Prospects

### 7.1 Frontiers in nanotechnology breakthroughs

Nanotechnology has driven innovations across various fields, including medicine, food, and the environment. In sepsis treatment, it has enhanced precision and personalization through intelligent nanomaterials that respond to environmental factors such as pH, temperature, and magnetic fields, enabling adaptive drug delivery and improving treatment efficacy [45]. The integration of artificial intelligence and gene editing further optimizes nanocarrier design, expanding their drug-carrying capacity and broadening applications for sepsis and other complex diseases.

Layer-by-Layer (LbL) assembly technology has revolutionized nanocarrier research by providing precise control over the surface properties of nanomaterials, catering to diverse biomedical needs [382]. Additionally, 3D printing has enabled personalized treatment by controlling material porosity and structure, fostering cell growth within scaffolds and advancing tissue engineering and regenerative medicine [383].

Nanobiotechnology has significantly improved functional molecule development. Through ligand design and intelligent regulation, bioavailability of bioactive substances like catechins, tea polyphenols, and theanine has been enhanced [384]. When incorporated into biomedical nanomaterials, these substances gain increased stability in complex biological environments, expanding their potential for disease treatment and health promotion.

Next-generation nanomaterials, driven by advancements in synthetic technology, offer improved biocompatibility and specialized functions. Using LbL assembly, nanomaterials can be designed with specific functions and directional properties. For example, red blood cell-loaded nanoparticles show promise in in vivo imaging and tumor photothermal therapy due to their biodegradability and safety [385]. Lipid-based nanomaterials and those with specific geometric shapes, such as cubes, also excel in drug delivery applications due to their superior performance [386].

In the food sector, nanotechnology is revolutionizing the use of preservatives. As demand for clean food and natural additives rises, natural preservatives modified with nanotechnology improve antimicrobial efficacy while maintaining desirable physicochemical and sensory properties [387]. Nanocapsulation, with its high surface-to-volume ratio, addresses challenges in food quality and safety and is widely used in food additives and anti-spoilage packaging [388]. However, large-scale production and process optimization remain challenges. Technological standardization can enhance product consistency and add significant economic value. For instance, Prolamin-based composite particles optimize particle interactions to improve drug loading efficiency, release rates, and colloidal stability, offering a framework for developing new materials [389,390].

### 7.2 Clinical Application Potential

Nanomaterials encapsulating bioactive substances hold great potential for personalized medicine, particularly in treating sepsis. They enable efficient drug delivery while minimizing damage to healthy tissues and reducing side effects. Multi-omics analyses, such as transcriptomics and proteomics, are crucial for understanding the interactions between nanomaterials and the body, providing insights into adaptive responses and toxicity mechanisms [314,391,392]. Fluorescence quenching technology is also widely used to study drug-nanomaterial interactions, particularly their accessibility with serum albumin, aiding in drug design and delivery optimization [393].

Animal models that closely mimic human physiology are essential for evaluating the safety, toxicity, and pharmacokinetics of exosome-based nanotherapies. These models provide critical data supporting the clinical feasibility of nanomedicines [394]. Using the image-based prediction modeling framework, it is possible to achieve accurate planning of personalized drug delivery in cancer treatment by analyzing patients' imaging data to predict the distribution and efficacy of drugs in vivo [395]. Additionally, the use of green resources for nanoparticle synthesis offers cost-effective, environmentally friendly alternatives. Plant-based nanoparticles, such as gold/silver oxides, are emerging as promising candidates for commercial use due to their simple synthesis and excellent biocompatibility [396].

Future research should focus on clarifying the roles of plant materials in nanoparticle synthesis, such as their function as reducing agents or carriers. This will optimize the design of plant-based nanomaterials and enhance their synergistic effects. Bioactive compounds in plant-based diets show potential in reducing chronic disease incidence, but further studies on cellular signaling, bioavailability, and synergistic effects are needed to expand their therapeutic functions [397]. The rapid development of 3D printing technology presents new opportunities for personalized treatment. By integrating bioactive substances with nanomaterials or other carriers through low-temperature, solvent-free processes, 3D printing enables precise design of drug delivery systems, enhancing treatment specificity and flexibility [398]. With growing interest in natural products, bioactive compounds from plant-based foods are increasingly recognized for their medical applications. Nanotechnology enhances their bioavailability, improving both clinical and commercial use. For instance, nanoliposomes can encapsulate both hydrophilic and lipophilic molecules, showing promise in dietary interventions and disease prevention. Plant-based nanovesicles also offer new avenues for developing nutritional supplements and functional foods [399]. Nanotechnology has emerged as a promising approach in food additives and packaging to prevent spoilage. However, to fully realize its potential, challenges related to process optimization and large-scale production must be overcome. Establishing standardized practices will not only enhance food industry outcomes but also have a substantial impact on the global economy [400].

### 7.3 Interdisciplinary collaboration and global trends

The integration of nanomedicine, immunology, and exercise physiology is advancing sepsis treatment by combining precise drug delivery, immune modulation, and metabolic effects. This interdisciplinary approach is expanding therapeutic possibilities and shaping multidimensional treatment strategies. Nanotechnology also offers solutions for environmental remediation, including pollutant treatment and ecological impact mitigation. For example, the synthesis of nanoparticles from microalgae and *T. ornata* enhances their stability and therapeutic properties [401,402]. In cancer therapy, Nisin-PLA-PEG-PLA nanoparticle formulations show promise in targeting gastrointestinal, liver, and blood cancers. Green-synthesized AgNPs, like those from Cyperus rotundus, have demonstrated biocompatibility, antibacterial efficacy, and wound healing potential [403]. Additionally, nanoparticles from turmeric and Artemisia annua have shown promise in treating inflammatory diseases and cancer [404]. Interdisciplinary collaboration in nanomedicine, immunology, and exercise physiology is uncovering new disease mechanisms and advancing personalized treatments.

## 8 Conclusion

Nanomaterials encapsulating biochemicals hold significant potential for sepsis treatment by enhancing antimicrobial, antioxidant, and immune-modulatory therapies. However, challenges such as nanomaterial toxicity, standardization issues, and clinical translation barriers remain. Future research should prioritize optimizing the safety and controllability of these nanomaterials to facilitate their clinical application in sepsis treatment. Particular attention should be given to the following directions: First, developing low-toxicity, biodegradable nanomaterials to minimize potential adverse effects; second, establishing standardized protocols for nanomaterial synthesis and characterization to ensure reproducibility and reliability; and third, conducting large-scale clinical trials to validate the efficacy and safety of nanomaterial-encapsulated bioactive substances in diverse patient populations. Additionally, interdisciplinary collaboration between nanotechnology, immunology, and clinical medicine will be crucial to advancing the field and overcoming existing challenges.

## CRediT authorship contribution statement

**Zhiwei Li:** Writing – review & editing, Writing – original draft, Resources, Methodology, Investigation, Formal analysis, Data curation. **Bin Luo:** Writing – review & editing, Writing – original draft, Visualization, Software, Resources, Methodology. **Yisheng Chen:** Writing – review & editing, Writing – original draft, Resources, Project administration, Formal analysis, Data curation. **Lingling Wang:** Writing – review & editing, Writing – original draft, Validation, Software, Resources, Methodology, Data curation. **Yezi Liu:** Writing – review & editing, Writing – original draft, Validation, Software, Resources, Formal analysis. **Jintong Jia:** Writing – review & editing, Writing – original draft, Resources, Methodology, Formal analysis. **Mengsi Chen:** Writing – review & editing, Writing – original draft, Methodology, Investigation, Formal analysis. **Shuting Yang:** Writing – review & editing, Writing – original draft, Validation, Resources, Formal analysis. **Haojun Shi:** Writing – review & editing, Writing – original draft, Formal analysis, Data curation. **Lihua Dai:** Writing – review & editing, Writing – original draft, Resources, Data curation. **Lei Huang:** Writing – review & editing, Writing – original draft, Validation, Project administration, Formal analysis. **Changmin Wang:** Writing – review & editing, Writing – original draft, Validation, Methodology, Formal analysis, Data curation. **Jia Liu:** Writing – review & editing, Writing – original draft, Supervision, Formal analysis, Data curation.

## Funding

This research did not receive any specific grant from funding agencies in the public, commercial, or not-for-profit sectors.

## Declaration of competing interest

The authors declare that they have no known competing financial interests or personal relationships that could have appeared to influence the work reported in this paper.

## Data Availability

Data will be made available on request.
